# Single-cell analysis of transcriptome and DNA methylome in human oocyte maturation

**DOI:** 10.1371/journal.pone.0241698

**Published:** 2020-11-05

**Authors:** Bo Yu, Naresh Doni Jayavelu, Stephanie L. Battle, Jessica C. Mar, Timothy Schimmel, Jacques Cohen, R. David Hawkins

**Affiliations:** 1 Department of OBGYN, University of Washington School of Medicine, Seattle, Washington, United States of America; 2 Institute for Stem Cell and Regenerative Medicine, University of Washington, Seattle, Washington, United States of America; 3 Departments of Medicine and Genome Sciences, University of Washington, School of Medicine, Seattle, Washington, United States of America; 4 Department of Systems and Computational Biology, Albert Einstein College of Medicine, Bronx, New York, United States of America; 5 Reprogenetics LLC, Livingston, New Jersey, United States of America; University of Bonn, Institute of Experimental Hematology and Transfusion Medicine, GERMANY

## Abstract

Oocyte maturation is a coordinated process that is tightly linked to reproductive potential. A better understanding of gene regulation during human oocyte maturation will not only answer an important question in biology, but also facilitate the development of *in vitro* maturation technology as a fertility treatment. We generated single-cell transcriptome and used our previously published single-cell methylome data from human oocytes at different maturation stages to investigate how genes are regulated during oocyte maturation, focusing on the potential regulatory role of non-CpG methylation. *DNMT3B*, a gene encoding a key non-CpG methylation enzyme, is one of the 1,077 genes upregulated in mature oocytes, which may be at least partially responsible for the increased non-CpG methylation as oocytes mature. Non-CpG differentially methylated regions (DMRs) between mature and immature oocytes have multiple binding motifs for transcription factors, some of which bind with *DNMT3B* and may be important regulators of oocyte maturation through non-CpG methylation. Over 98% of non-CpG DMRs locate in transposable elements, and these DMRs are correlated with expression changes of the nearby genes. Taken together, this data indicates that global non-CpG hypermethylation during oocyte maturation may play an active role in gene expression regulation, potentially through the interaction with transcription factors.

## Introduction

Proper development of the mature oocyte is an essential part of reproduction and the prerequisite for fertilization and downstream embryonic development. In humans, oocytes are arrested in the prophase of meiosis I and remain quiescent until decades later when the follicles are recruited for growth [1]. Oocytes must mature and undergo transcriptional and physiological changes in preparation for ovulation and fertilization. When the arrested prophase I oocyte (also known as germinal vesicle or GV stage) is recruited to grow and mature, the germinal vesicle breaks down and the oocyte resumes meiosis I, leading to the intermediate maturation state metaphase I (MI stage). As meiosis II progresses, the oocyte divides into a polar body and a mature metaphase II oocyte (MII stage). The MII oocyte is ready for ovulation and fertilization, and carries the maternal genome into the embryo after fertilization. The gene regulatory dynamics associated with oocytes maturation are of great interest to developmental biology and for clinical development of better *in vitro* maturation technology as a fertility treatment.

The transcriptomic landscape of oocyte development has been described in several mammalian species including cow, rabbit, rhesus, mouse and human [2, 3]. From these studies we have learned that there are fewer transcripts in the MII oocyte which can be due to both reduced expression and RNA degradation [2, 4]. Thousands of differentially expressed genes, both upregulated and downregulated, have been identified from GV to MII [2, 4, 5]. Similarly, previous DNA methylation studies on developing oocytes have provided great insight to the epigenomic landscape of the maturing oocyte. Mouse studies have shown that there is an overall increase in DNA methylation as oocytes grow and mature [6, 7]. In humans, the process of DNA methylome erasure in primordial germ cells and in pre-implantation embryos was recently shown in several genome-wide studies, although many of these studies focused on embryo development rather than oocyte maturation [8, 9]. Our group published data in single-cell DNA methylome of human oocytes at various maturation stages, and demonstrated genome-wide increase in non-CpG methylation as oocytes mature [10]. However, few previous studies have investigated the epigenomic regulatory mechanisms that control differential gene expression during human oocyte maturation. Further elucidation of gene regulatory mechanisms would help us understand the transcriptomic dynamics of oocyte maturation.

Here, we generated single-cell mRNA-seq data and correlated with single-cell whole genome bisulfite sequencing (WGBS) data from the same individual cohort at three human oocyte maturation stages (GV, MI, MII), in order to gain a better understanding of gene regulation during human oocyte maturation through DNA methylation. We find that the accumulation of non-CpG methylation in mature MII oocytes is accompanied by upregulation of the *DNMT3B* gene. Distal differentially methylated regions (DMRs) are negatively correlated with gene expression and gene body DMRs positively correlating with gene expression. We identify transcription factors (TFs), such as ETS1 and YY1, which have binding motifs at DMRs and potentially direct DNMT3B methylation in the maturing oocyte genome. Many of the DMRs are located in the regulatory regions of the genome. These results suggest a regulatory role of non-CpG methylation in transcriptomic changes during oocyte maturation.

## Results

### Gene expression, DEGs and pathways

Oocytes at three maturation stages (immature GV and MI stages, and mature MII stage) were collected from 17 women of reproductive age using assisted reproductive technologies. We generated single-cell transcriptome and single-cell DNA methylome data in these oocytes (S1 Table). Some of the individuals contributed oocytes of two maturation stages or to both datasets. In total, 21 mRNA-seq libraries were generated from single oocytes and analyzed together with previously published DNA methylome data on 32 single-cell WGBS libraries [10].

For transcriptome data processing, mRNA sequencing reads were mapped to hg19 and Cufflinks [11] was used to calculate FPKM values (S2 Table). Individual samples were assessed for quality and uniformity based on their distribution of FPKM values and high reproducibility was observed among samples within the same maturation stage (S1A and S1B Fig in S1 File). We found high correlation between FPKM and normalized read count (Pearson correlation values for GV are 0.88, MI are 0.86 and MII are 0.88) and therefore decided to continue all downstream analysis with FPKM values. We calculated the pairwise Pearson’s correlation coefficient to assess intra- versus inter-individual variation (S1C Fig in S1 File). Since we observed high inter-individual correlations within the same maturation stage similar to intra-individual correlations, we merged samples of the same stage and used the merged FPKM values for all further analyses.

**Fig 1:**
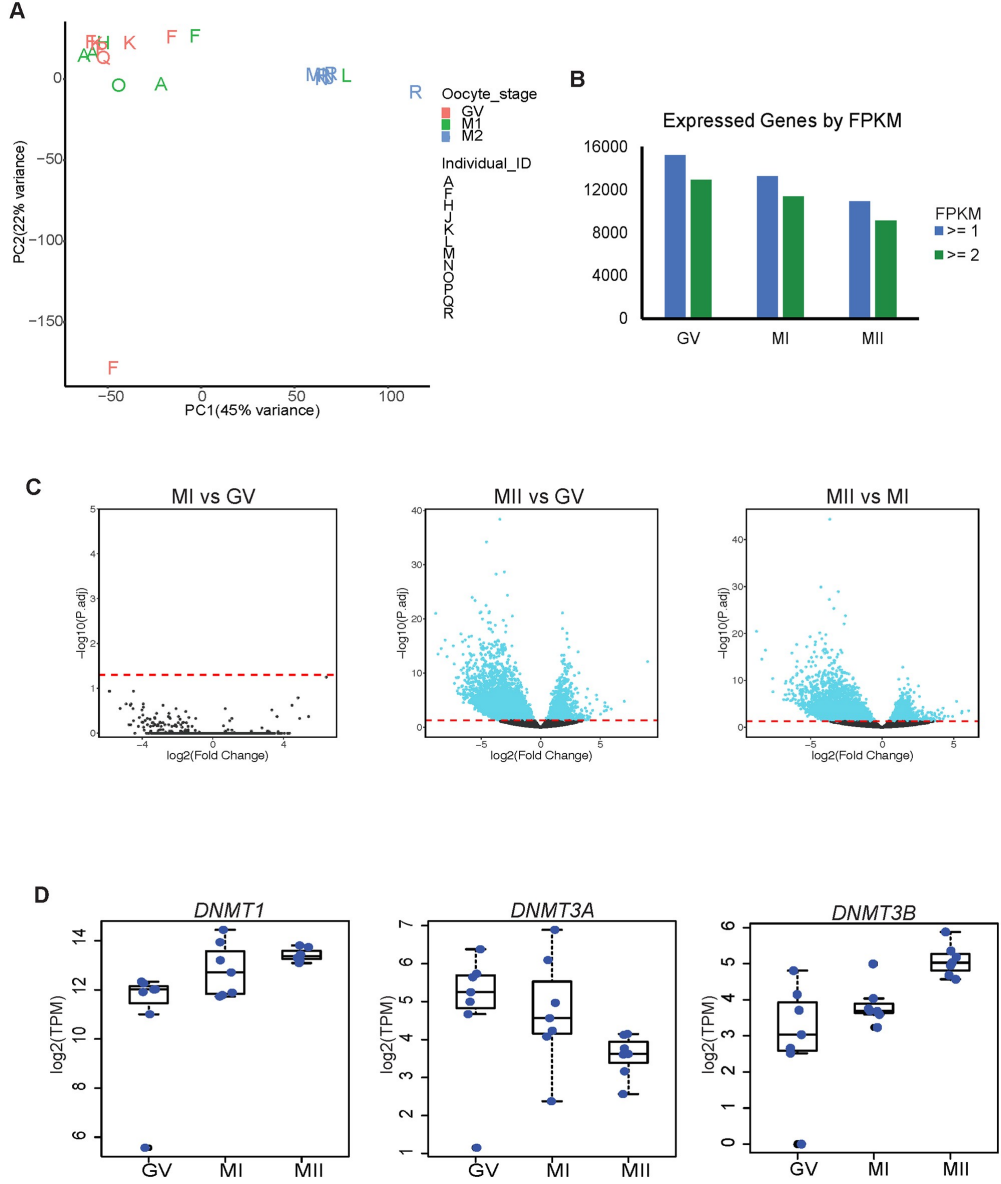
Gene expression in GV, MI and MII oocytes. A. PCA of top 1000 expressed transcripts in individual RNA-Seq libraries. Letters on plot correspond to individual sample IDs and color corresponds to oocyte stage. B. Bar chart of total number of transcripts expressed at equal to or less than 1 or 2 FPKM cutoffs from merged single-cell RNA-Seq datasets. C. Volcano plot of differential gene expression in pairwise comparisons. Dashed line is adjusted p-value cutoff of 0.05. D. Bar chart of TPM values of DNMTs. Each blue dot is the TPM value of each individual sample.

We first asked whether the oocyte maturation stages could be uniquely identified based on their transcriptome. Principal component analysis of the top 1000 expressed genes shows a clear separation between the immature GV or MI oocytes and the mature MII oocytes, with the GV and MI stages being almost indistinguishable from each other (Fig 1A). We next looked at the global gene expression patterns in each maturation stage. A total of 15,224, 13,283 and 10,892 genes were expressed in GV, MI, and MII oocytes, respectively (FPKM > = 1; Fig 1B). Few genes were specifically expressed in a single stage (427 GV, 3 MI and 7 MII specific genes) (S3 Table). The very low numbers of cell type specific genes in the later stages of maturation suggests that *de novo* transcription occurs in the earliest stage of oocyte maturation [12, 13]. As expected, all four zona pellucida glycoprotein genes (*ZP1*, *ZP2*, *ZP3*, and *ZP4*) were highly expressed in all three stages (S1D Fig in S1 File). We were also able to validate oocyte specific genes *DAZL*, *GDF9*, and *BMP15* as being expressed in all maturation stages and *RBBP7* as being significantly highly expressed in MII oocytes compared to MI (log2 fold change 1.44; S1E Fig in S1 File) [14–16] (S1D Fig in S1 File). *PTTG1* and *TUBB8* were among the highest expressed genes in MII oocytes (S1D Fig in S1 File). *PTTG1* encodes a securin protein that prevents chromosome segregation [17]. *TUBB8* encodes the beta-tubulin subunit primarily expressed in oocytes and the early embryo. Mutations in *TUBB8* have been identified in infertile women with oocyte maturation arrest [18–20]. These genes have clear relevance and significance in oocyte maturation and development.

We used DESeq2 [21] to calculate the number of differentially expressed genes (DEGs) in a pairwise manner among the three oocyte maturation stages. A large number of DEGs was observed between MII oocytes and the two immature stages (Fig 1C). There were 5,979 DEGs (defined by adjusted p-value ≤ 0.05 and fold change ≥ 2) between MII and GV and 4,835 between MII and MI. There were no DEGs between MI and GV, supporting the results of our PCA analysis that these two oocyte stages have similar gene expression at the level of protein coding genes. This was also previously observed in microarray data on human oocytes [5, 13, 22]. In order to ensure our findings were not due to a bias in our analysis, we repeated the DEG analysis using a different program, edgeR [23]. Even though edgeR called fewer DEGs, there was a high degree of overlap with those called by DESeq2 (S1E Fig in S1 File).

Of the DEGs we identified using DESeq2, 1,362 were upregulated in MII compared to GV and 4,617 were downregulated. In the MII/MI pairwise comparison, 1,077 were upregulated in MII and 3,758 were downregulated. We focused on the MII/MI DEGs as these two timepoints mark the transition from immature to mature oocyte (S4 Table). The upregulated genes fell into pathways involving RNA degradation, splicing and transport (Table 1). As expected, cell cycle, ubiquitin mediated proteolysis and oocyte meiosis were also significantly enriched pathways. Genes involved in RNA dynamics are anticipated to be upregulated in MII oocytes given that: (1) the oocyte accumulates maternal RNAs necessary for early embryonic development, (2) there is slow maternal RNA degradation during oocyte maturation, as detected by the reduction in transcripts, and (3) maternal RNA degradation occurs shortly after fertilization [3]. Downregulated pathways included various metabolic pathways including the TCA cycle and oxidative phosphorylation, which was consistent with previous observations of alternative glucose metabolism pathways during oocyte cytoplasmic maturation [24–27].

**Table 1:** KEGG pathways.

MII Upregulated DEGs	
Pathway	Adj p-value
Ribosome	1.85E-07
RNA degradation	0.0000112
Spliceosome	0.0001546
Cell cycle	0.0001546
RNA transport	0.00652
Ubiquitin mediated proteolysis	0.01526
Oocyte meiosis	0.03
MII Downregulated DEGs	
Pathway	Adj p-value
Metabolic pathways	6.98E-08
Oxidative phosphorylation	5.72E-07
Parkinson’s disease	0.0002435
Carbon metabolism	0.001298
2-Oxocarboxylic acid metabolism	0.001929
Huntington’s disease	0.003961
Pyrimidine metabolism	0.004119
Non-alcoholic fatty liver disease	0.004944
Proteasome	0.004944
Biosynthesis of amino acids	0.006568
SNARE interactions in vesicular transport	0.01449
Alzheimer’s disease	0.01509
Glyoxylate and dicarboxylate metabolism	0.01581

Listed are significantly enriched KEGG pathways for up-and downregulated DEGs (differentially expressed genes) in the MII to MI pairwise comparison.

We queried our RNA-seq data for expression of epigenetic regulators (S1F Fig in S1 File and Fig 1D). *TET3* is significantly upregulated in MII oocytes with a log2 fold change of 3.3 compared to MI oocytes. *TET3* and *TET2*, which are expressed in all three stages of oocyte maturation, are important in removing methylation in the zygotic genome upon fertilization [28, 29]. *DNMT1* is highly expressed in all three oocyte maturation stages and *DNMT3B* is significantly upregulated in MII compared to MI oocytes (log2 fold change 2.8). *DNMT3A* expression is reduced in MII and there is no detectable *DNMT3L*. *DNMT3B* encodes a key *de novo* DNA methyltransferase which has the ability to methylate non-CpG sites [30]. The upregulation of *DNMT3B* led us to take a closer look at the correlation between DNA methylation, especially non-CpG methylation, and gene expression in the maturing oocyte.

### DNA methylation correlates with gene expression in oocytes

**Fig 2:**
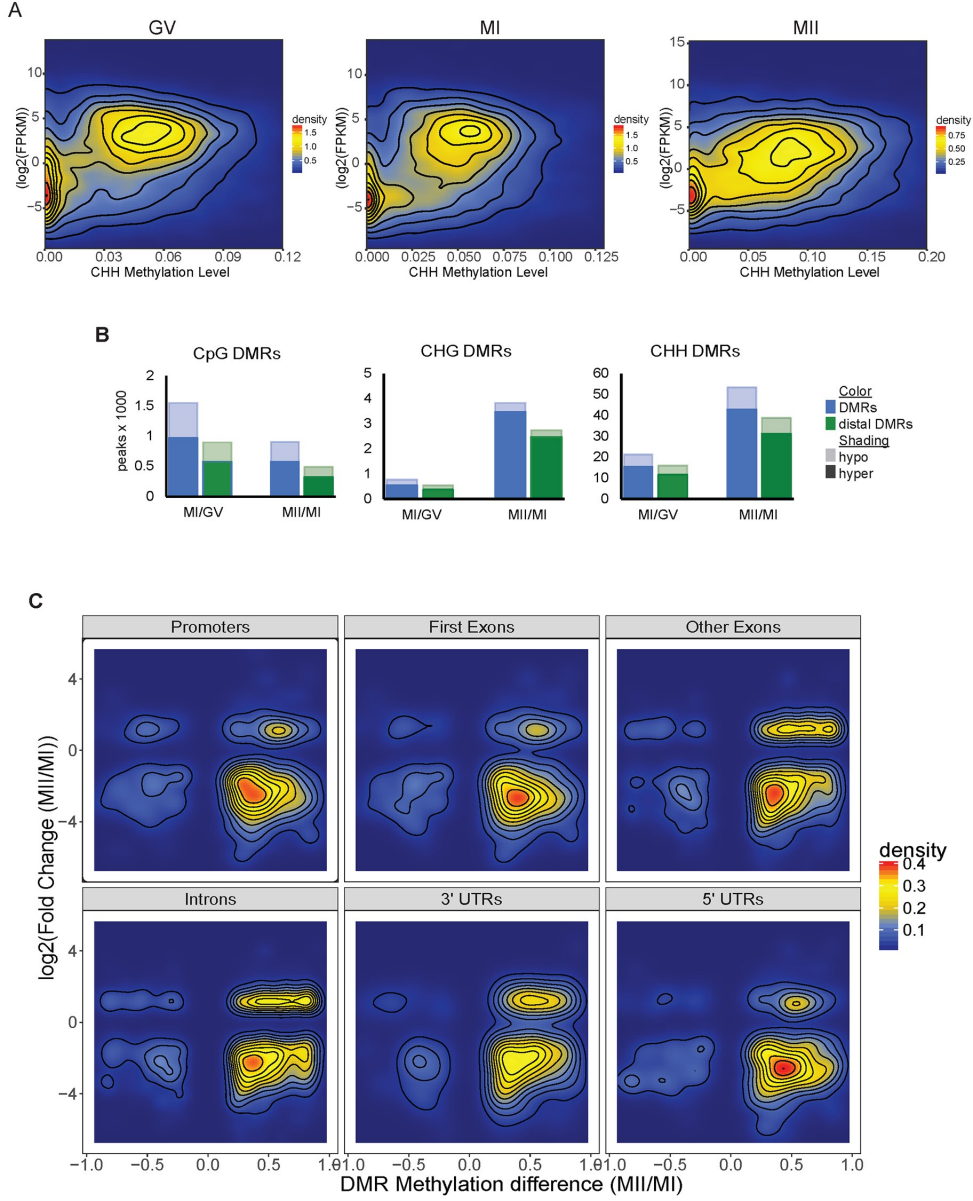
Gene body methylation and expression correlations. A. Density plots of gene body methylation levels for CHH context and the corresponding gene’s expression level in GV, MI and MII oocytes. Density color scale to the right of each respective plot. B. Differentially methylated region (DMR) counts by the thousands for each C context in GV, MI and MII oocytes. Blue bar to the left is total DMR count. Green bar to the right is the count of distal DMRs. Hypermethylated DMRs are solid color while hypomethylated DMRs are lightly shaded. C. Methylation level of CHH DMRs in MI/MII comparison in specific genic regions and their corresponding gene expression level. Scale for density plot is to the right.

We previously described global DNA methylation trends during human oocyte maturation [10] and observed similar average CpG methylation levels across all three stages while the non-CpG average methylation level nearly doubles in the MII stage compared to the immature oocytes (S2A Fig in S1 File). Now with the transcriptomic data, we found that the increase of non-CpG methylation in the MII stage coincides with the upregulation of *DNMT3B* in MII. There is a weak positive correlation between gene body methylation and gene expression for all cytosine contexts in all three stages (Fig 2A and S2B Fig in S1 File). This correlation is stronger for methylated CpGs, which have higher average methylation levels than non-CpGs.

Differentially methylated regions (DMRs) have been proposed to be *cis*-regulatory elements in other cell types [31], therefore, we then investigated the correlation between DMRs (S5 Table) and gene expression changes during oocyte maturation. We asked how many of the DMRs are distal, not overlapping a gene promoter (-2kb to +500bp of gene TSS), exons or untranslated regions (UTRs). These distal DMR have the greatest potential to be distal regulatory elements. Between 70–75% of all non-CpG DMRs are distal (S6 Table), whereas only 57% of MI/GV CpG DMRs and 54% of MII/MI CpG DMRs are distal. Over 80% of all distal DMRs overlap with ENCODE DNase I hypersensitivity sites (S6 Table). The majority of DMRs were hypermethylated in the more mature stage in the pairwise comparisons to the immature stages (Fig 2B and S6 Table). Most hypermethylated DMRs in MII oocytes were associated with downregulation of gene expression in the MII/MI comparison (Fig 2C and S2C Fig in S1 File). However, some hypermethylated CHH DMRs, specifically in introns, exons and 3’-UTRs, are associated with upregulated gene expression (Fig 2C). This is consistent with our observation that 603 (60%) of the upregulated MII/MI DEGs have a DMR in their gene body.

### DNMT3B binding partners and hypermethylation of gene targets

**Fig 3:**
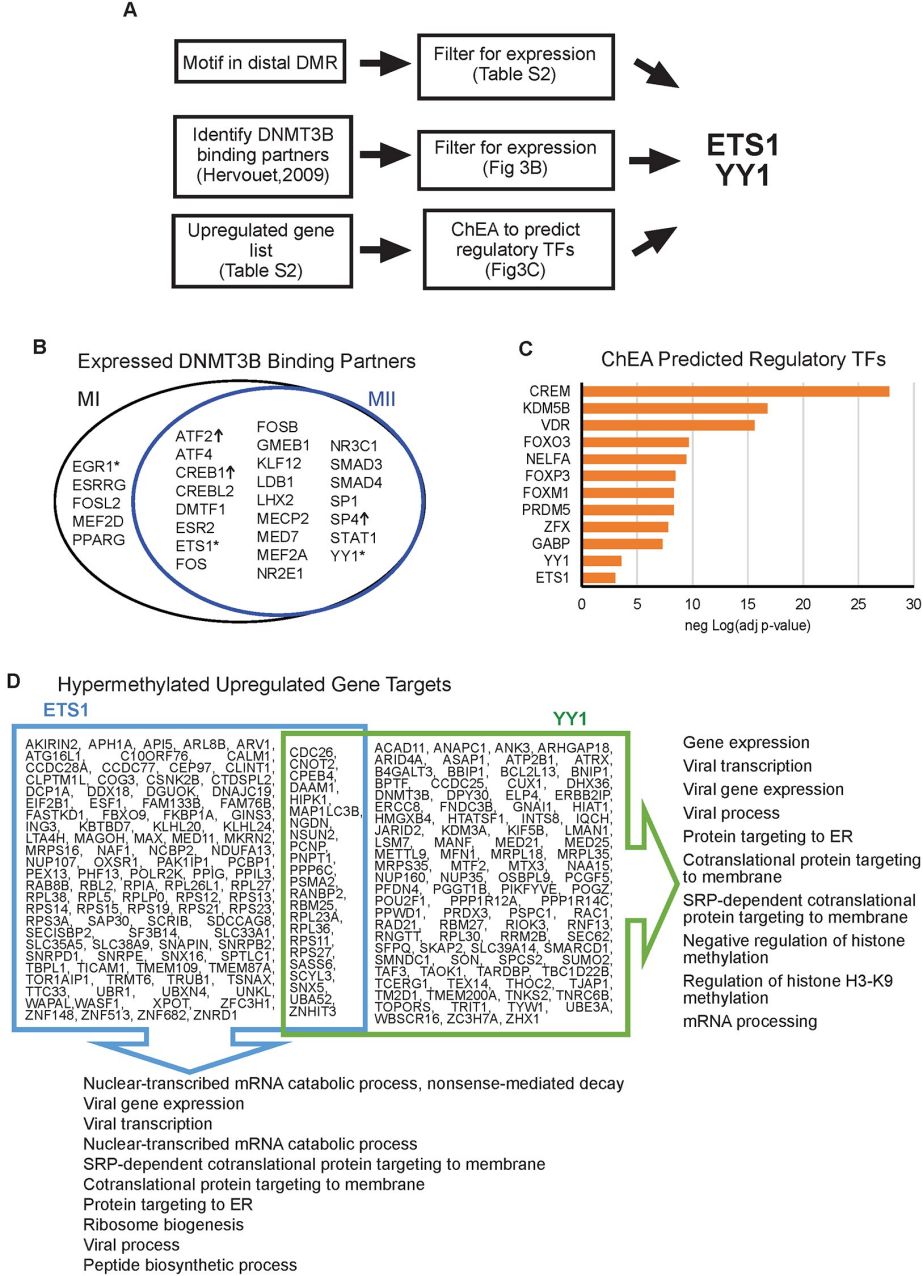
Regulatory transcription factors at differentially methylated regions and their gene targets. A. Flow chart of the two different approaches that identified ETS1 and YY1 as regulatory transcription factors (TF) in oocytes. B. Venn diagram showing DNMT3B binding partners that are expressed in MI or MII (FPKM > = 1). Of the expressed transcription factors (TFs), only three (denoted with “*”) have their binding motifs present in MII-to-MI DMRs. TF with “↑” are significantly upregulated in MII compared to MI. C. Bar charts of adjusted p-value of select TFs predicted to regulate MII-to-MI downregulated genes by ChEA. Orange bars have adjusted p-value less than 0.05. D. MII-to-MI downregulated genes predicted to be regulated by ETS1 and YY1. The top ten Gene Ontology (GO) Biological Processes for each gene list are displayed. ER: endoplasmic reticulum.

Of the DNMTs, only *DNMT3B* is upregulated as oocytes mature. DNMT3B is one of the *de novo* methyltransferases and can methylate non-CpG motifs [30]. We took several different approaches in an attempt to identify what factors may physically direct DNMT3B to methylate regions in the maturing oocyte genome (Fig 3A). First, we looked for transcription factors (TF) that have been previously shown to directly bind DNMT3B via protein-protein binding array [32]. Of the DNMT3B-TF binding partners, 29 were expressed in MI or MII stage oocytes (Fig 3B). Expression of three of DNMT3B-TF binding partners were significantly upregulated in MII compared to MI: *ATF2*, *CREB1* and *SP4*. Next, we looked for transcription factor binding motifs in distal MII/MI DMRs (S7 Table). Of the expressed TFs, only three (EGR1, ETS1 and YY1, denoted with * in Fig 3B) have their binding motifs present in MII/MI DMRs, and only ETS1 and YY1 have their known motif in an MII distal DMR.

In a complementary approach, we ran our list of upregulated genes through ChIP Enrichment Analysis (ChEA) [33] which predicts regulatory TFs from a gene list (Fig 3A). ETS1 and YY1 were predicted to be regulatory TFs by ChEA with statistically significant enrichment scores (adjusted p-value = 9.0*10^−4^ and 2.9*10^−4^, respectively; Fig 3C). Notably, we also found that ETS1 (ChEA adjusted p-value 5.3*10^−17^) was predicted to be a regulatory TFs in the downregulated gene list as well. Therefore, we took a closer look at ETS1 and YY1 and their gene targets in MII stage oocytes.

ETS1 is a TF involved in many cellular processes including tumorigenesis and hematopoietic stem cell development [34, 35]. ETS family proteins are phosphorylated by MAP kinases and their activity as either an activator or repressor is modulated by co-factor binding [34]. YY1 is a ubiquitous transcription factor shown to have both activator and repressor activity [36, 37]. YY1 can interact with other proteins to stabilize enhancer-promoter loops [37, 38]. More importantly, YY1 is required for oocyte growth and follicular expansion [39]. Mice with a conditional knockout of YY1 are infertile and lack mature oocytes [39]. Using ChEA data, we identified the upregulated gene targets of ETS1 and YY1 (Fig 3D). There were 123 gene targets of ETS1 and 117 gene targets of YY1 that were upregulated in MII compared to MI. Common Gene Ontology (GO) Biological Processes between the two gene lists included mRNA processes, viral gene expression and protein targeting particularly to the ER. YY1 regulated genes were also involved in epigenetic regulation, specifically of histone H3 K9 methylation. Additionally, there are 603 genes upregulated in MII with a DMR (either CpG or non-CpG) in their gene body. Of these genes, 62 had an ETS1 binding site in their DMR and 151 genes had an YY1 binding site, with 60 genes in common between these two groups. We found these gene body DMRs to be hypermethylated in 58 of the 62 genes with ETS1 and 140 of the 151 genes with YY1 binding sites. These data suggest that the increased non-CpG methylation in mature MII oocytes may not be a generalized random event, but rather at least partially be directed by the binding of DNMT3B with TFs such as ETS1 and YY1, and the resulting gene body hypermethylation at these TF binding sites is associated with upregulation of these targeted genes.

### LINE1 elements at DMRs

**Fig 4:**
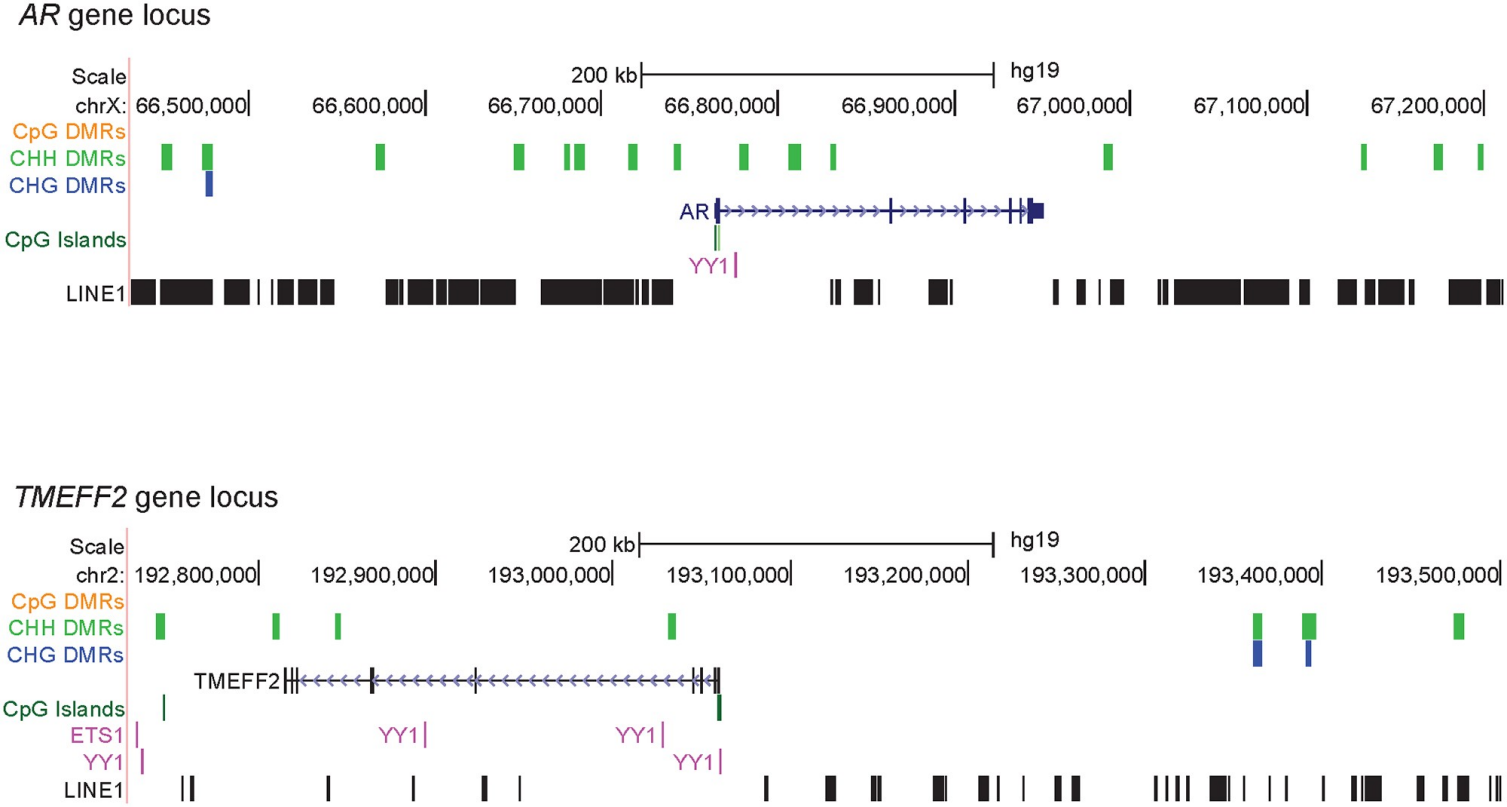
Genes regulated by hypermethylated non-CpG DMRs at LINE1 elements. UCSC browser images of the *AR* and *TMEFF2* loci. Hypermethylated DMRs for each context are shown and the location of LINE1 elements are also displayed.

Transposable elements (TEs) have been shown to function as regulatory elements and are expressed in the oocyte [8, 40–42]. Since TEs have been shown to be hypermethylated in MII oocytes compared to pre-implantation embryo [8], we asked if there was differential methylation at TEs during oocyte maturation. We used TE coordinates from RepeatMasker to assess how many DMRs were at TEs in the genome. Over 95% of all DMRs, and over 98% of non-CpG DMRs, were at transposable elements in the genome (S4 Table). We focused further on LINE1 elements as those have been shown to be expressed in mammalian oocytes and early embryogenesis [42, 43]. We used GREAT (Genome Regulatory Architecture Tools) [44] to predict biological processes that were related to the genes located in the vicinity of the LINE1 elements overlapping MII hypermethylated non-CpG distal DMRs (S3 Fig in S1 File). A key gene involved in multiple processes was the *AR* gene (Fig 4). The *AR* gene is expressed in GV oocytes and its expression drops to undetectable in MII oocytes. AR is important for germinal vesicle breakdown when the GV oocyte resumes meiosis to become the mature MII oocyte [45]. *AR* knockout mice have many phenotypes associated with reduced fertility [45]. *TMEFF2* is another gene located in close proximity to the region with overlapping LINE1 elements and non-CpG DMRs (Fig 4). *TMEFF2* is upregulated in early oocyte development in the primordial/primary follicle stage [46] and in our data we observed downregulation of TMEFF2 from GV to MII. These are two examples in which hypermethylated LINE1 is associated decreased expression of nearby genes. Interestingly, ETS1 and YY1 binding sites were also found in these regions.

## Discussion

We generated single-cell mRNA-seq data and correlated with our recently published WGBS data in human oocytes at three stages of maturation, in order to gain understanding of potential *cis*-regulatory roles of DNA methylation, especially non-CpG methylation on gene expression during oocyte maturation. We observed less RNA transcripts and general downregulation of gene expression as the oocyte matures. However, there are genes that are specifically upregulated in mature MII oocytes and these include epigenetic regulators. The upregulation of *DNMT3B* is accompanied by the increase in non-CpG methylation in the mature MII genome. Most of the MI/MII DMRs, particularly non-CpG DMRs, exist in the regulatory region of the genome such as LINE1 elements. Previously it has been shown that LINE1 activity in GV oocytes is essential for progression to the MII stage [43]. We observe several TF binding motifs in the MI/MII DMRs of which, ETS1 and YY1 have previously been shown to interact with DNMT3B and a subset of their targeted genes showed hypermethylated gene body and upregulated gene expression in MII oocytes. Interestingly, mice with a conditional knockout of YY1 are infertile and lack mature oocytes [39]. These data indicate that some gene expression changes during oocyte maturation may be associated with non-CpG hypermethylation which may be directed by DNMT3B-TF binding. Validation experiments directly demonstrating the binding of ETS1 and YY1 in conjunction with DNMT3B at a subset of the target genes in human oocytes would be a valuable future direction when single-cell binding assays become available.

Previous studies in mouse and human oocytes, brain tissue, and pluripotent stem cells [7, 8, 10, 47, 48] found non-CpG methylation to be a prominent feature in these cell types. In oocyte development, the increase of non-CpG methylation is specific to maturing oocytes [49]. In mice, newborn non-growing oocytes are mostly depleted of non-CpG methylation but gain it as cells enter the GV stage [7]. The establishment of non-CpG methylation in the immature stages in mice is dependent on Dnmt3a and Dnmt3L activity, while Dnmt3b was dispensable as shown in mutant mouse studies [7]. While we do not detect expression of *DNMT3L* in human oocytes, *DNMT3A* is upregulated in the immature stages. There is a switch from *DNMT3A* to *DNMT3B* expression once oocytes enter the mature MII stage. This is accompanied by an increase in non-CpG methylation. It is possible that the decrease in *DNMT3A* transcripts from immature to mature oocytes is due to its increased translation, and DNMT3A, together with DNMT3B, contributes to the *de novo* methylation of non-CpG sites in the maturing oocytes. After all, knockdown of DNMT3A and DNMT3B in human embryonic stem cell lines was previously shown to result in a global reduction of non-CpG methylation [47]. Non-CpG methylation was also shown to be exclusively catalyzed by Dnmt3a and Dnmt3b in embryonic cell lines [30], drosophila [50], and more recently in human mitochondrial DNA [51, 52].

The regulatory role that CpG methylation plays in gene expression is well established, while the functional role of non-CpG methylation is poorly understood. In general, if CpG sites located in a gene body are methylated, gene expression is increased [53, 54]. On the other hand, if CpG sites located in promoters or enhancers are methylated, transcriptional silencing occurs because these regions become heterochromatic and are not bound by transcription activators [55–57]. These expected patterns of correlation between CpG methylation and gene expression or repression were also observed in non-CpG methylation in our data (Fig 2A and S2B Fig in S1 File). Interestingly, the increase in non-CpG methylation as oocytes mature is associated with downregulation of gene expression in most genomic contexts. However, some of the increases of non-CpG methylation in the gene body are associated with increased gene expression, and this positive correlation is seen in introns, exons, and 3’-UTRs (Fig 2C). During the maternal-zygotic transition in early embryo development, maternal RNA degradation is induced by the binding of proteins and microRNAs to the 3'-UTR of target RNAs [58, 59]. DNA methylation is one of the regulatory mechanisms in controlling microRNA expressions [60, 61]. Whether increased non-CpG methylation in mature oocytes play any role in the transcription of those microRNAs that interact with 3’-UTR and regulate maternal-zygotic transition remains to be investigated.

Intragenic gene regulation via DNMT3B has been observed in other cell-types. For example, mutations in DNMT3B occur in the majority of patients with Immunodeficiency, Centromere instability and Facial anomalies (ICF) syndrome. In these patients, intragenic binding of DNMT3B was shown to affect expression of transcript isoforms [62]. Gene repression via DNMT3B-TF directed methylation has been observed in other cell types. Dnmt3B interacts with the repressor E2F6 to methylate promoters and silence germline genes in murine somatic tissues [63]. DNMT3B can also interact with PU.1 to methylate promoters of genes during monocyte to osteoclast differentiation [64]. We observed significant upregulation of three of DNMT3B-TF binding partners from the transition of immature to mature oocytes: *ATF2*, *CREB1* and *SP4*. All three transcription factors are crucial in embryo and fetal development. The AP-1 family transcription factor ATF2 is essential for development and tissue maintenance in mammals. In bovine oocytes, the protein levels of ATF2 remained constant from GV stage oocytes throughout embryo development, suggesting its role in early embryo development [65]. ATF2 is required for central nervous system development in mouse embryos and loss of ATF2 function leads to motoneuron degeneration in the brain [66]. Creb1 and its related Creb family member Atf1 are essential for normal preimplantation embryo development. Deletion of *Creb1* and *Atf1* results in the loss of viability and failure of normal development of peri-implantation mouse embryos [67]. Spatial and temporal expression of *Crebs* is important for final oocyte maturation and may also regulate oocyte growth in fish [68]. Mice with null mutation in *Sp4* gene exhibited a high mortality rate after birth, growth retardation, pronounced delay in sexual maturation, male infertility and underdeveloped uteri in females, indicating the essential role of Sp4 in growth, puberty and reproduction [69].

DNMT3B recruitment to genomic loci may also alter the chromatin structure as DNMT3B has been shown to co-immunoprecipitate with HDAC1 and HDAC2 in human and mouse cell lines [70]. One of the TFs we identified in our study YY1, is essential for oocyte maturation and is known to act as either an activator or a repressor through interaction with other regulatory proteins [37, 39]. Our data suggests that DNMT3B recruitment by TFs, such as ETS1 and YY1, may contribute to targeted gene regulation in the MII oocyte.

In summary, we conducted single-cell genome-wide study of the transcriptome and DNA methylome in human oocytes at three stages of maturation (GV, MI, and MII). The data will serve as an important resource for other germ cell and stem cell researchers due to the rare availability of human mature oocytes and the single-cell approaches. Our analysis provides insights into the potential *cis*-regulatory role of non-CpG methylation on gene expression in human oocyte maturation.

## Materials and methods

### Oocyte collection

All human oocytes used in this study were obtained in embryology laboratories at Saint Barnabas Center for Reproductive Medicine and Sher Institutes for Reproductive Medicine under the regulatory oversight of Institutional Review Board (IRB)-approved Human Subjects protocol at each institution. After oocyte retrieval procedures under standard Assisted Reproductive Technology protocols, oocytes that were destined to be discarded were collected under previously obtained written informed consent. All consented materials were donated anonymously and carried no personal identifiers. None of the oocytes was exposed to sperms or discarded due to fertilization or quality issues. GV and MI oocytes were collected at the time of maturity check. Most of the MII oocytes were collected from oocyte donors who had excess oocytes to dispose, due to rare circumstances that removed them from the donor list.

To eliminate contamination from cumulus cells and other cell types, we removed zona pellucida from each oocyte using either acid Tyrode’s solution or mechanical separation techniques. Each oocyte was washed in PBS 2–3 times and immediately frozen in 2 μL of PBS in a −80°C freezer until shipment on dry ice. Oocytes were received for further studies at Albert Einstein College of Medicine (AECOM) under the approval of AECOM IRB, which deemed the project exempt under 45 CRF 46.102(f). All oocytes were processed by the same embryologist (T.S.), including the removal of zona pellucida, washing, cryopreserving, and shipping. All single-cell WGBS and mRNA-seq experiments were carried out by the same individual (B.Y.).

### Library preparation and mRNA-seq

From each single oocyte, cDNA was synthesized using SMART-Seq v4 ultra low input RNA kit (Clontech, cat # 634889). An mRNA-seq library was then prepared from each sample using standard Illumina TruSeq protocol. All libraries were multiplexed and sequenced on the same flow cell in order to minimize sequencing batch effect. Sequencing was performed at the Epigenomics and Genomics Shared Facility at Albert Einstein College of Medicine.

### Gene expression data analysis

The sequence data was aligned to the human genome build 19 using the STAR aligner with default parameters. Expression level of individual mRNA-seq data was calculated in TPM using RSEM [71]. Merged gene expression values were computed in FPKM units using Cufflinks [11]. Differentially expressed genes were calculated using DESeq2 [21] and edgeR [23, 72]. Only DEseq2 calculated differentially expressed genes were used for further analysis.

Gene lists were put into Enrichr [73, 74] to obtain Gene Ontology terms, KEGG pathways, ChEA and ENCODE Transcription factor ChIP-seq enrichment scores.

### WGBS and differentially methylated regions analysis

WGBS data was taken from Yu et al., 2017 [10], where methods and equations are described in depth. Briefly, raw sequence reads were trimmed for adapter contamination using Trim Galore! and mapped to hg19 using Bismark. Lambda phage DNA spike-ins were used to calculate bisulfite conversion efficiency. All conversion efficiencies were above 98%. Methylation levels were called using Bismark Methylation Extractor and methylation levels were calculated for each context (CpG, CHG, CHH) separately. Differential methylated regions were calculated using a 3 kb sliding window across the genome with a 600 bp step size. Overlapping regions were merged for each context separately.

BEDTools v2.24.0 [75] was used for all genomic features comparisons. Gencode v19 was used for genic annotations and transposable element coordinates were taken from RepeatMasker in the UCSC Genome Browser. HOMER [76] was used to identify Transcription factor binding motifs in regions of interest. Transcription factor binding sites were obtained from the ENCODE data track on UCSC Genome browser (http://genome.ucsc.edu/cgi-bin/hgTrackUi?db=hg19&g=wgEncodeRegTfbsClusteredV3). LINE1 elements at non-CpG hypermethylated differentially methylated regions were put into GREAT tool to predict gene targets.

## Supporting information

S1 Table Donor information. Related to Fig 1. The 17 donors from which single cell whole genome bisulfite sequencing (WGBS) or single cell mRNA-seq data was generated. The number of oocytes used for each method is written as: number of oocytes used for WGBS, number of oocytes used for mRNA-seq. A “-”indicates no oocytes were used for that method. A blank cell indicates no oocytes from that stage were used. There is no sample E.
(DOCX)

S2 Table RNA-seq raw sequencing data. Related to Fig 1. Shown is the total number of sequencing reads and duplication level of each library/sample used in this study for RNA-seq. Reads were trimmed for poor quality and Illumina sequencing adapter.
(DOCX)

S3 Table List of genes specifically expressed in each maturation stage. GV: Germinal Vesicle stage; MI: Metaphase I stage; MII: Metaphase II stage.
(XLSX)

S4 Table List of Differentially Expressed Genes (DEGs) in MI/MII comparison. (XLSX)

S5 Table Lists of Differentially Methylated Regions (DMRs) in MI/MII comparison. Three tabs: CpG, CHH, CHG.
(XLSM)

S6 Table Differentially methylated region stats. Related to Fig 2. Statistics on differentially methylated regions (DMRs). (DHS: DNase I hypersensitive sites).
(DOCX)

S7 Table Expressed TFs with binding motifs in DMRs. Related to Fig 3. Transcription factor (TF) motifs found in MII/MI differential methylated regions (DMRs).
(DOCX)

S1 File (PDF)
