# Supplementary material for: Single-cell analysis of transcriptome and DNA methylome in human oocyte maturation
Source: PLoS One. 2020 Nov 5;15(11):e0241698. doi: 10.1371/journal.pone.0241698 (PMC7643955; doi:10.1371/journal.pone.0241698)
Supplement: S1 Table — Related to Fig 1. The 17 donors from which single cell whole genome bisulfite sequencing (WGBS) or single cell mRNA-seq data was generated. The number of oocytes used for each method is written as: number of oocytes used for WGBS, number of oocytes used for mRNA-seq. A “-”indicates no oocytes were used for that method. A blank cell indicates no oocytes from that stage were used. There is no sample E. (DOCX) [file pone.0241698.s001.docx]

Donor Samples WGBS, mRNA-seq

| Patient ID (age) | GV | MI | MII |
| --- | --- | --- | --- |
| A (32 yo) | 2, - | 3, 3 |  |
| B (33 yo) | 3, - | 1, - |  |
| C (37 yo) |  | 1, - |  |
| D (39 yo) |  | 1, - | 1, - |
| F (38 yo) | 3, 3 | -, 1 |  |
| G (41 yo) | 1, - |  |  |
| H (35 yo) |  | 1, 1 |  |
| I (28 yo) | 1, - |  |  |
| J (38 yo) |  |  | 3, 1 |
| K (41 yo) | 1, 2 | 1, - |  |
| L (34 yo) | 1, - | 2, 1 |  |
| M (28 yo) |  |  | 3, 1 |
| N (27 yo) |  |  | 3, 2 |
| O (36 yo) |  | -,1 |  |
| P (31 yo) | -, 1 |  |  |
| Q (40 yo) | -, 1 |  |  |
| R (31 yo) |  |  | -, 3 |

**Table S1. Donor Information. Related to Figure 1**

The 17 donors from which single cell whole genome bisulfite sequencing (WGBS) or single cell mRNA-seq data was generated. The number of oocytes used for each method is written as: number of oocytes used for WGBS, number of oocytes used for mRNA-seq. A “-“ indicates no oocytes were used for that method. A blank cell indicates no oocytes from that stage were used. There is no sample E.
