# Supplementary material for: Single-cell analysis of transcriptome and DNA methylome in human oocyte maturation
Source: PLoS One. 2020 Nov 5;15(11):e0241698. doi: 10.1371/journal.pone.0241698 (PMC7643955; doi:10.1371/journal.pone.0241698)
Supplement: S2 Table — Related to Fig 1. Shown is the total number of sequencing reads and duplication level of each library/sample used in this study for RNA-seq. Reads were trimmed for poor quality and Illumina sequencing adapter. (DOCX) [file pone.0241698.s002.docx]

| Sample | Patient ID | Oocyte Stage | Total Reads Sequenced | Duplication level | Reads after trimming | Transcripts detected (log2(TPM) >= 1) |
| --- | --- | --- | --- | --- | --- | --- |
| 1 | A | MI | 11,164,256 | 38.41% | 11,158,983 | 13,743 |
| 2 | A | MI | 27,960,729 | 59.36% | 27,946,776 | 9,786 |
| 3 | A | MI | 14,052,560 | 37.29% | 14,046,825 | 13,900 |
| 4 | F | GV | 25,987,999 | 49.6% | 25,972,524 | 13,341 |
| 5 | F | MI | 16,821,201 | 51.1% | 16,813,065 | 9,999 |
| 6 | F | GV | 9,761,183 | 43.78% | 9,755,708 | 10,733 |
| 7 | F | GV | 19,464,789 | 39.92% | 19,455,676 | 10,024 |
| 8 | H | MI | 16,476,702 | 39.69% | 16,469,324 | 13,745 |
| 9 | J | MII | 11,141,192 | 44.71% | 11,134,274 | 10,776 |
| 10 | K | GV | 11,004,353 | 41.66% | 10,999,123 | 12,733 |
| 11 | K | GV | 11,309,635 | 40.81% | 11,304,216 | 13,319 |
| 12 | L | MI | 9,817,670 | 46.7% | 9,813,579 | 10,095 |
| 13 | M | MII | 9,717,810 | 46.45% | 9,711,572 | 11,002 |
| 14 | N | MII | 8,503,595 | 47.13% | 8,498,815 | 10,572 |
| 15 | N | MII | 7,800,795 | 50.35% | 7,794,364 | 10,316 |
| 16 | O | MI | 21,194,291 | 43.85% | 21,183,798 | 13,518 |
| 17 | P | GV | 17,561,145 | 38.77% | 17,547,732 | 13,806 |
| 18 | Q | GV | 17,416,638 | 36..43% | 17,407,200 | 14,155 |
| 19 | R | MII | 10,300,610 | 47.82% | 10,295,683 | 10,602 |
| 20 | R | MII | 12,100,394 | 49.39% | 12,095,147 | 10,644 |
| 21 | R | MII | 10,475,975 | 59.32% | 10,470,461 | 7,810 |

**Table S2. RNA-seq Raw Sequencing Data. Related to Figure 1**

Shown is the total number of sequencing reads and duplication level of each library/sample used in this study for RNA-seq. Reads were trimmed for poor quality and Illumina sequencing adapter.
