# Supplementary material for: Single-cell analysis of transcriptome and DNA methylome in human oocyte maturation
Source: PLoS One. 2020 Nov 5;15(11):e0241698. doi: 10.1371/journal.pone.0241698 (PMC7643955; doi:10.1371/journal.pone.0241698)
Supplement: S6 Table — Related to Fig 2. Statistics on differentially methylated regions (DMRs). (DHS: DNase I hypersensitive sites). (DOCX) [file pone.0241698.s006.docx]

| % TE overlap | 97% | 99% | 99% |  | 96% | 99% | 99% |
| --- | --- | --- | --- | --- | --- | --- | --- |
| % distal DMR DHS overlap | 92% | 85% | 84% |  | 93% | 86% | 85% |
| % distal | 57% | 70% | 75% |  | 54% | 71% | 73% |
| Hypo | 585 | 244 | 5,982 |  | 330 | 365 | 10,546 |
| Hyper | 974 | 545 | 15,471 |  | 575 | 3,469 | 42,923 |
| DMRs | 1,559 | 789 | 21,453 |  | 905 | 3.834 | 53,469 |
| Comparison | MI v GV | MI v GV | MI v GV |  | MII v MI | MII v MI | MII v MI |
| Context | CpG | CHG | CHH |  | CpG | CHG | CHH |

**Table S6. Differentially Methylated Region Stats. Related to Figure 2**

Statistics on differentially methylated regions (DMRs).

(DHS: DNase I hypersensitive sites)
