# Supplementary material for: Single-cell analysis of transcriptome and DNA methylome in human oocyte maturation
Source: PLoS One. 2020 Nov 5;15(11):e0241698. doi: 10.1371/journal.pone.0241698 (PMC7643955; doi:10.1371/journal.pone.0241698)
Supplement: S7 Table — Related to Fig 3. Transcription factor (TF) motifs found in MII/MI differential methylated regions (DMRs). (DOCX) [file pone.0241698.s007.docx]

| **Transcription Factor** | **Consensus Sequence** | **Log P-value** |
| --- | --- | --- |
| **CpG Hyper** | | |
| IRF1 | ACTTTCGTTTCT | -4.608 |
| **CHG Hyper** | | |
| ETS1 | MACAGGAAGT | -5.669 |
| **CHG Hypo** | | |
| NPAS2 | KCCACGTGAC | -5.921 |
| STAT3 | CTTCCGGGAA | -5.283 |
| MAX | RCCACGTGGYYN | -4.78 |
| **CHH Hyper** | | |
| NFKB1 | GGGGGAATCCCC | -19.51 |
| EGR1 | TGCGTGGGYG | -19.16 |
| YY1 | CAAGATGGCGGC | -16.39 |
| ZBTB33 | GGVTCTCGCGAGAAC | -16.14 |
| SMAD2 | AGGTGHCAGACA | -12.26 |
| MAX | RCCACGTGGYYN | -12.18 |
| NFYA | RGCCAATSRG | -9.78 |
| ETS1 | MACAGGAAGT | -9.07 |
| EBF1 | GTCCCCWGGGGA | -7.15 |
| CLOCK | GHCACGTG | -6.65 |
| EBF1 | DGTCCCYRGGGA | -5.84 |
| NR1H4 | AGGTCANTGACCTB | -4.88 |
| **CHH Hypo** | | |
| MAX | RCCACGTGGYYN | -7.276 |
| NR1H4 | AGGTCANTGACCTB | -6.570 |
| NFKB1 | GGGGGAATCCCC | -5.701 |
| NFYA | RGCCAATSRG | -5.686 |

**Table S7. Expressed TFs with binding motifs in DMRs. Related to Figure 3**

Transcription factor (TF) motifs found in MII/MI differential methylated regions (DMRs).
